# Thermoelectric Energy Micro Harvesters with Temperature Sensors Manufactured Utilizing the CMOS-MEMS Technique

**DOI:** 10.3390/mi13081258

**Published:** 2022-08-05

**Authors:** Yi-Xuan Shen, Yao-Chuan Tsai, Chi-Yuan Lee, Chyan-Chyi Wu, Ching-Liang Dai

**Affiliations:** 1 Department of Mechanical Engineering, National Chung Hsing University, Taichung 402, Taiwan; 2 Department of Bio-Industrial Mechatronics Engineering, National Chung Hsing University, Taichung 402, Taiwan; 3 Smart Sustainable New Agriculture Research Center (SMARTer), Taichung 402, Taiwan; 4 Department of Mechanical Engineering, Yuan Ze Fuel Cell Center, Yuan Ze University, Taoyuan 320, Taiwan; 5 Department of Mechanical and Electro-Mechanical Engineering, Tamkang University, New Taipei 251, Taiwan

**Keywords:** thermoelectric energy micro harvester, complementary metal oxide semiconductor, microelectromechanical system, thermocouple, cooling sheet

## Abstract

This study develops a TEMH (thermoelectric energy micro harvester) chip utilizing a commercial 0.18 μm CMOS (complementary metal oxide semiconductor) process. The chip contains a TEMH and temperature sensors. The TEMH is established using a series of 54 thermocouples. The use of the temperature sensors monitors the temperature of the thermocouples. One temperature sensor is set near the cold part of the thermocouples, and the other is set near the hot part of the thermocouples. The performance of the TEMH relies on the TD (temperature difference) at the CHP (cold and hot parts) of the thermocouples. The more the TD at the CHP of the thermocouples increases, the higher the output voltage and output power of the TEMH become. To obtain a higher TD, the cold part of the thermocouples is designed as a suspended structure and is combined with cooling sheets to increase heat dissipation. The cooling sheet is constructed of a stack of aluminum layers and is mounted above the cold part of the thermocouple. A finite element method software, ANSYS, is utilized to compute the temperature distribution of the TEMH. The TEMH requires a post-process to obtain the suspended thermocouple structure. The post-process utilizes an RIE (reactive ion etch) to etch the two sacrificial materials, which are silicon dioxide and silicon substrate. The results reveal that the structure of the thermocouples is completely suspended and does not show any injury. The measured results reveal that the output voltage of the TEMH is 32.5 mV when the TD between the CHP of the thermocouples is 4 K. The TEMH has a voltage factor of 8.93 mV/mm^2^K. When the TD between the CHP of the thermocouples is 4 K, the maximum output power of the TEMH is 4.67 nW. The TEMH has a power factor of 0.31 nW/mm^2^K^2^.

## 1. Introduction

Thermoelectric energy micro harvesters (TEMHs) that are able to convert wasted heat into electric power are applied in various devices and systems [1], such as self-powered wireless devices [2], wearable devices [3], electronic devices [4], and health monitoring and tracking systems [5]. For instance, Leonov [6] developed a wearable electrocardiography system in which the power was supplied by 8 TEMHs. A wearable watch with a TEMH was presented by Torfs [7], where the TEMH was utilized to generate power from human body heat. Bavel [8] presented the used of TEMHs to supply a wireless electroencephalography headband for detecting brain signals.

Microelectromechanical system (MEMS) technology has employed microfabrication and micromachining to develop various micro-devices [9,10,11,12] and micro-transducers [13,14,15,16,17]. There were many thermoelectric energy micro harvesters made utilizing this technology. For instance, Su [18] developed a TEMH manufactured by utilizing the microfabrication and bonding techniques. The TEMH was composed of p-type and n-type SiGe thermocouples, in which the total number of thermocouples was 1300. The hot part of the thermocouple was made on a silicon substrate, and the cold part of the thermocouple was made on another silicon substrate. Both the silicon substrates were bonded together using the bonding technique, and the thermocouples formed a cubic structure. The height of the legs for the thermocouple was 6 μm. The TEMH had an output voltage of 0.7 V when the TD (temperature difference) of the CHP (cold and hot parts) for the thermocouple was 50 °C. Peng [19] employed MEMS technology to develop a TEMH that contained eight thermoelectric cells. Each thermoelectric cell, designed in a circular shape, was composed of 25 thermocouples. The materials of the thermocouples were n-type polysilicon and p-type polysilicon. The measured results presented that the TEMH had an output voltage factor of 0.178 mV/mm^2^K and a power factor of 1.47 × 10^−3^ pW/mm^2^K^2^. A TEMH with test structures, proposed by Zhang [20], was fabricated utilizing MEMS technology. In order to improve thermoelectric gathering and obtain a better performance, a metal heat sink was combined with the TEMH. The metal heat sink was put at the center of the TEMH. The output voltage factor and the power factor of the TEMH were 0.58 V/cm^2^K and 2.76 × 10^−2^ μW/cm^2^k^2^, respectively. Noyan [21] proposed a TEMH which was fabricated utilizing silicon micromachining technology. The material of the thermocouples for the TEMH was SiGe nanowire that was deposited by a chemical vapor deposition on a micro-platform. The test results presented that the SiGe nanowire TEMH could harvest 7.1 μW/cm^2^ at a waste heat temperature of 200 °C. Xie [22] utilized a CMOS process to fabricate a TEMH with a heat sink layer. The heat sink layer, made of aluminum and amorphous silicon, was deposited on the cold part of the TEMH, and it was able to enhance heat dissipation at the cold part of the TEMH. The thermocouples of the TEMH were constructed of n-type polysilicon and p-type polysilicon. The area of the TEMH was 1 cm^2^. The measured results presented that the power factor of the TEMH was 0.052 μW/cm^2^K^2^. Yang [23] design an in-plane TEMH and made the TEMH using a CMOS process. The in-plane TEMH was established by thermocouples with n-type polysilicon and p-type polysilicon. To reduce electrical resistance and heat loss, the optimal dimensions of the thermocouple were analyzed. The legs of the thermocouple were 60 μm long and 2 μm wide. The area of the TEMH was 1.2 × 1.2 mm^2^. The results showed that the power factor of the TEMH was 0.0473 μW/cm^2^K^2^, and its output voltage factor was 4.423 V/cm^2^K. Sun [24] manufactured a micro-power generator that was a hybrid of a TEMH and a photoelectric generator utilizing a CMOS process. The thermocouples of the TEMH consisted of n-type polysilicon and p-type polysilicon legs, which had a length of 75 μm and a width of 20 μm. The test results showed that the output voltage factor of the TEMH was 0.316 V/cm^2^K, and the power factor of the TEMH was 6.34 × 10^−3^ μW/cm^2^K^2^. The photoelectric generator had an efficiency of 4.11%. A flexible TEMH, presented by Glatz [25], was fabricated utilizing the photolithography and electrochemical deposition processes. The materials of the thermocouples for the TEMH were Ni and Cu, which were deposited on a flexible polymer substrate utilizing electroplating. The measured results presented that the power factor of the flexible TEMH was 0.83 μW/cm^2^K^2^. Huesgen [26] utilized a combination of bulk and surface micromachining processes to create a TEMH. The legs of the thermocouples for the TEMH were aluminum and n-type polysilicon. The hot part of thermocouples was fabricated as a suspended structure that could reduce the heat sink and increase thermoelectric gathering. The results showed that the TEMH had a power factor of 3.63 × 10^−3^ μW/mm^2^K^2^. Another flexible TEMH, developed by Glatz [27], was manufactured employing the electrochemical deposition process. The thermoelectric materials of the flexible TEMH were p-type and n-type Bi_2_Te_3_, which were electroplated on a polymer substrate. The test results presented that the power factor of the flexible TEMH was 0.29 μW/cm^2^K^2^. Wang [28] employed MEMS technology to create a TEMH. The thermoelectric materials for the TEMH were p-type and n-type Bi_2_Te_3_ nanowire arrays. The electrochemical deposition was utilized to coat a Bi_2_Te_3_ nanowire array on a porous alumina template. The TEMH was designed as a laminar structure. The p-type Bi_2_Te_3_ nanowire array had a Seebeck coefficient of 260 μV/K and the n-type Bi_2_Te_3_ nanowire array had a Seebeck coefficient of −188 μV/K. Kao [29] proposed a TEMH made utilizing a commercial CMOS process. The TEMH consisted of 24 thermocouples, which were made of n-type polysilicon and p-type polysilicon. The results showed that the power factor of the TEMH was 6.4 × 10^−2^ nW/cm^2^K^2^. Table 1 summarizes the performances of the TEMHs.

The CMOS process is usually utilized to manufacture IC (integrated circuit) components. Recently, this process has not only been used to fabricate IC, but also MEMS devices. The approach that uses the CMOS process to make MEMS devices is called the CMOS-MEMS technique [30,31,32,33]. There have been various micro-devices [34,35,36] developed by the technique. In this study, a TEMH with temperature sensors is designed and made utilizing the CMOS-MEMS technique. The temperature sensors are utilized to monitor the temperature of the CHP of the thermocouples. To enhance heat dissipation, the cold part of the thermocouples is designed as a suspended structure and combined with the cooling sheets. Micro-devices developed by the COMS-MEMS technique have the advantages of small volume, easy fabrication, and fast mass-production [37,38]. Compared to the MEMS technology, the CMOS-MEMS technique is easier to use in manufacturing micro-devices and achieving mass-production. These TEMHs [19,20,21,25,26,27,28] were fabricated using the MEMS technology. The fabrication of the TEMH in this work is easier than that of Peng [19], Zhang [20], Noyan [21], Glatz [25], Huesgen [26], Glatz [27], and Wang [28]. The power factor of the TEMH in this work exceeds that of Peng [19], Zhang [20], Sun [24], and Kao [29].

## 2. Design and Analysis of the Thermoelectric Energy Micro Harvester

**Figure 1:**
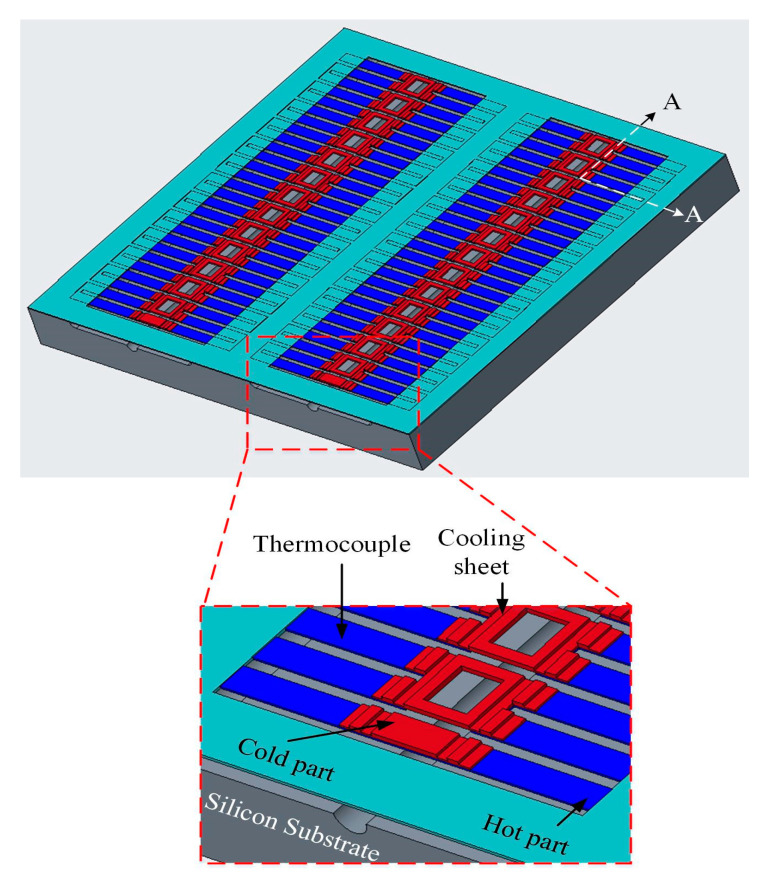
Structure for the TEMH (thermoelectric energy micro harvester).

Figure 1 shows the structure of the thermoelectric energy micro harvester with temperature sensors. The TEMH is designed based on the Seebeck effect. A series of 54 thermocouples is used to form the TEMH. Each thermocouple is constructed by matrial-1 and material-2 strips. Material-1 and material-2 are p-type polysilicon and n-type polysilicon, respectively. Thermocouples have a hot part and a cold part. The hot part of thermocouples is anchored on the silicon substrate, and the cold part of thermocouples is a suspended structure. In order to reduce the heat sink on the hot part of thermocouples, a thick silicon dioxide layer deposited on the hot part of the thermocouples because silicon dioxide is a low thermal conductivity material. To increase the heat sink on the cold part of the thermocouples, the cooling sheets are designed and set on the cold part of thermocouples. The working principle of the TEMH is that heat source inputs from the silicon substrate and conducts to the hot part of the thermocouples; then the heat source conducts to the cold part of the thermocouples and the cooling sheets. The heat source is dissipated by the cold part of thermocouples and the cooling sheets. When the thermocouples have a TD between the CHP, the TEMH generates an output voltage. To measure the temperature at the CHP of thermocouples in real time, the temperature sensors are designed and set near the cold parts and hot parts of the thermocouples. The TEMH area is 0.96 mm^2^. Each thermocouple has a length of 180 μm, a thickness of 0.2 μm, and a width of 26 μm.

**Figure 2:**
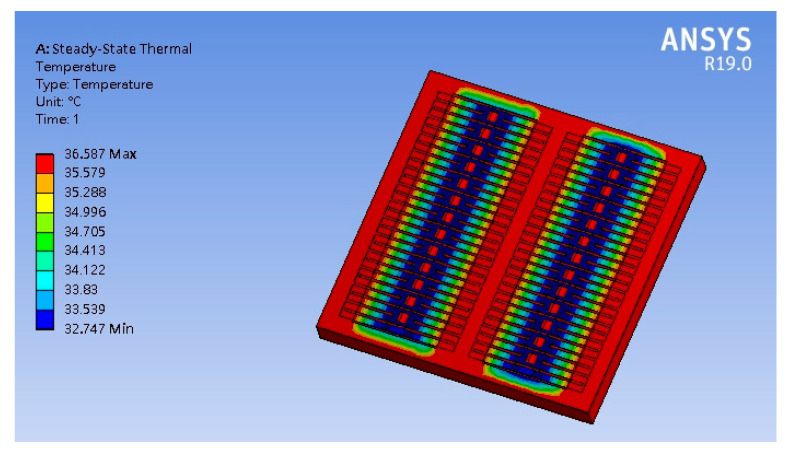
Computation of temperature distribution for the TEMH using the ANSYS.

The TD between the CHP of the thermocouples that deeply effect the output voltage and output power of the TEMH is an important factor for the TEMH. A finite element method software, ANSYS, is utilized to analyze the temperature distribution for the TEMH. First, it is necessary to construct the TEMH model according to the structure in Figure 1. The hot part of the thermocouples is located on the silicon substrate, and the cold part of the thermocouples is a suspended structure. Then, the TEMH model is meshed, and the triangular element is used to analyze the TEMH. There are about 45,000 elements generated. The boundary condition is that the hot part of the thermocouple is fixed. The material properties of the TEMH must be inputted into the ANSYS software. In the TEMH, the materials include silicon, aluminum, polysilicon, and silicon dioxide. Table 2 lists the thermal conductivity of the materials. The thermal conductivity of silicon is 150 W/m·K, and the thermal conductivity of aluminum is 236 W/m·K. The thermal conductivity of polysilicon is 31.5 W/m·K, and the thermal conductivity of silicon dioxide is 1.42 W/m·K. The thermal conductivity of these material is inputted into the ANSYS. The thermal flux is 30 pW/µm^2^K, which value is inputted into the ANSYS. Finally, the software carries out the computation for the temperature distribution of the TEMH. The computation of temperature distribution for the TEMH using the ANSYS is shown in Figure 2. The results present that the maximum temperature occurring at the hot part of the thermocouples is 36.587 °C, and the minimum temperature occurring at the cold part of the thermocouples is 32.747 °C. The CHP of the thermocouples have a TD of about 4 °C.

**Figure 3:**
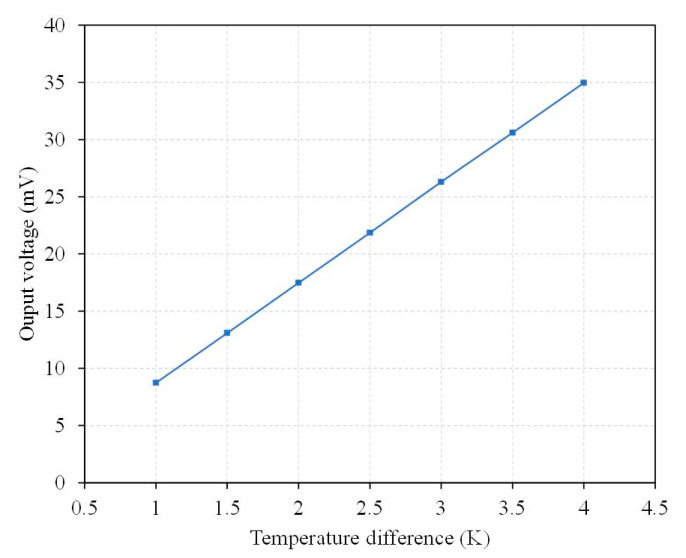
Evaluation of the output voltage for the TEMH.

The thermoelectric energy micro harvester produces an output voltage, as it has a TD between the CHP of the thermocouples. The output voltage of the TEMH is given by [39],

(1) $$V_{o}=m\left(\beta_{1}-\beta_{2}\right)\left(T_{ht}-T_{cd}\right)$$

where *m* is thermocouple number, *β*_1_ is the Seebeck coefficient of material-1, *β*_2_ is the Seebeck coefficient of material-2, *T_ht_* is the hot part temperature of the thermocouple, and *T_cd_* is the cold part temperature of the thermocouple. According to Equation (1), the output voltage of the TEMH relies on the thermocouple number, TD (*T_ht_* − *T_cd_*), and the Seebeck coefficient difference (*β*_1_ − *β*_2_). Increasing the thermocouple number could enhance the output voltage of the TEMH. The TD of the CHP of the thermocouple is proportional to the output voltage of the TEMH, so enhancing the TD could increase the TEMH output voltage. The Seebeck coefficient of the thermocouple materials is an important parameter. If both materials in the thermocouple show a large difference in the Seebeck coefficient, then the TEMH has a high output voltage. The Seebeck coefficient difference of thermocouples for the TEMH is 161 μV/K [23]. The value is substituted into Equation (1) to compute the output voltage for the TEMH. Figure 3 shows the output voltage for the TEMH under different TD. The results showed that the output voltage for the TEMH enhances as the temperature difference increases, and the output voltage for the TEMH is proportional to the TD between the CHP of the thermocouples. As shown in Figure 2, the output voltage for the TEMH changes from 8.7 mV at a TD of 1 K to 34.8 mV at a TD of 4 K. The evaluation of the output voltage factor for the TEMH is 9.4 mV/mm^2^K.

**Figure 4:**
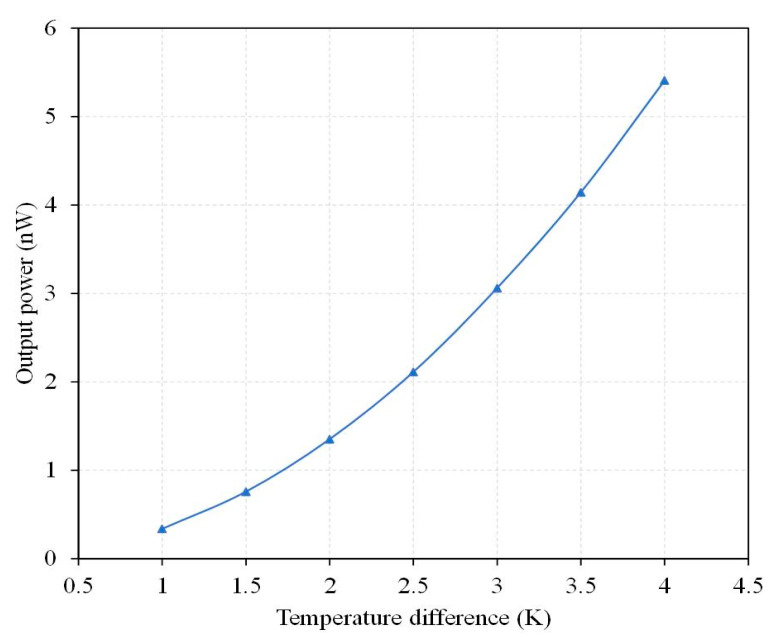
Evaluation of the maximum output power for the TEMH.

When the thermoelectric energy micro harvester connects with an external load resistance, the output power of the TEMH can be obtained. Suppose that the external load is equal to the internal resistance of the TEMH, then the maximum output power of the TEMH can be expressed as [40],

(2) $$P_{max}=\frac{V_{o}^{2}}{4R_{i}}$$

where *V_o_* represents the output voltage of the TEMH and *R_i_* is the internal resistance of the TEMH. According to Equation (2), the maximum output power of the TEMH relies on the output voltage and internal resistance of the TEMH. The greater the output voltage produced by the TEMH, the higher the maximum output power generated by the TEMH. Reducing the internal resistance of the TEMH could increase the maximum output power of the TEMH. To enhance the maximum output power of the TEMH, we design a low internal resistance for the thermoelectric energy micro harvester. The internal resistance of the thermoelectric energy micro harvester can be expressed as [25],

(3) $$R_{i}=\left(\frac{\rho_{1}l_{1}}{w_{1}t_{1}}+\frac{\rho_{2}l_{2}}{w_{2}t_{2}}\right)$$

where *ρ*_1_ is the resistivity of material-1 for the thermocouple, *l*_1_ is the length of material-1 for the thermocouple, *t*_1_ is the thickness of material-1 for the thermocouple, *w*_1_ is the width of material-1 for the thermocouple, *ρ*_2_ is the resistivity of material-2 for the thermocouple, *l*_2_ is the length of material-2 for the thermocouple, *t*_2_ is the thickness of material-2 for the thermocouple, and *w*_2_ is the width of material-2 for the thermocouple. In Equation (3), the first team (*ρ*_1_*l*_1_/*w*_1_*t*_1_) means the resistance of material-1 and the second team (*ρ*_2_*l*_2_/*w*_2_*t*_2_) means the resistance of material-2. The internal resistance of the TEMH relies on the resistivity, length, width, and thickness of material-1 and material-2 for the thermocouples. The more the resistivity of material-1 and material-2 for the thermocouple increases, the higher the internal resistance of the TEMH becomes. As the length of material-1 and material-2 for the thermocouple increases, then the internal resistance of the TEMH increases. Oppositely, as the thickness and width of material-1 and material-2 for the thermocouple reduce, the internal resistance of the TEMH increases. In the design for the thermoelectric energy micro harvester, there are 54 thermocouples. The dimensions of each thermocouple are explained as follows: the length of material-1 is 180 μm; the length of material-2 is 180 μm; the width of material-1 is 26 μm; the width of material-2 is 26 μm; the thickness of material-2 is 0.2 μm; and the thickness of material-2 is 0.2 μm. The number and dimensions of the thermocouples are substituted into Equation (3). The internal resistance of the TEMH is obtained, and the value is 56.5 kΩ. To analyze the maximum output power of the TEMH, Equation (2) is employed to evaluate the TEMH maximum output power. The output voltage of the TEMH in Figure 3 and the internal resistance of 56.5 kΩ are substituted into Equation (2). The maximum output power of the TEMH is obtained. The evaluation of the maximum output power for the thermoelectric energy micro harvester is shown in Figure 4. The results show that the maximum output power of the TEMH increases as the TD between the CHP for the thermocouple increases. The maximum output power for the TEMH is 0.34 nW at a TD of 1 K. When the TD increases to 4 K, the maximum output power becomes 5.41 nW. The evaluation of the power factor for the TEMH is 0.36 nW/mm^2^K^2^.

**Figure 5:**
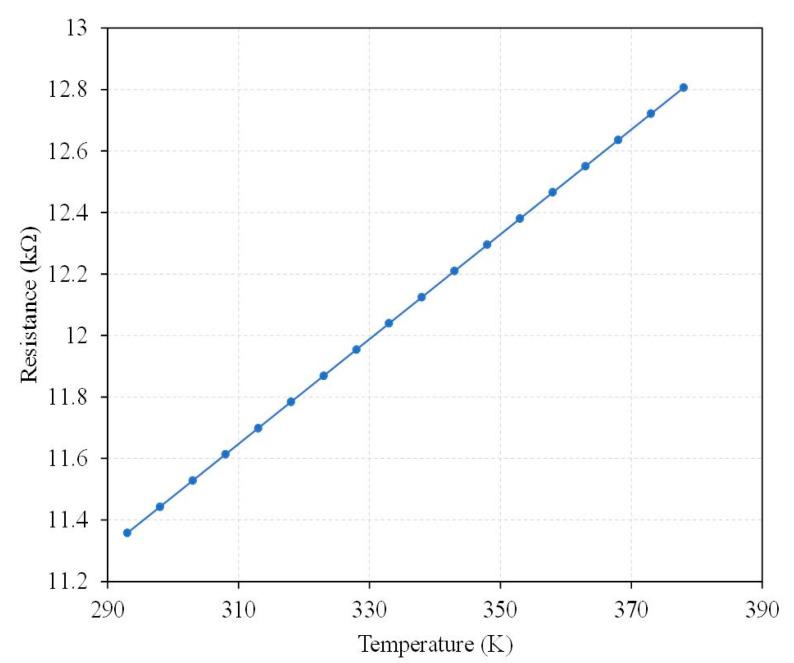
Evaluation of resistance change for the temperature sensor.

To monitor the temperature of the CHP for the thermocouples, the temperature sensors are designed. The temperature sensors are placed near the CHP for the thermocouples. The temperature sensors are the thermoresistive type. Polysilicon is used as the material for the temperature sensors because polysilicon has an excellent thermoresistive property. All the temperature sensors are made of a polysilicon strip and have the same dimensions, with a length of 1300 μm and a width of 0.95 μm. The initial resistance for the temperature sensor can be expressed as [41],

(4) $$R_{0}=R_{st}\frac{L_{st}}{W_{st}}$$

where *R_st_* is the sheet resistance for the temperature sensor, *L_st_* is the length for the temperature sensor, and *W_st_* is the width for the temperature sensor. According to Equation (4), the initial resistance of the temperature relies on the sheet resistance, length, and width of the polysilicon strip. The more the sheet resistance and length of the polysilicon strip increase, the higher the initial resistance of the temperature sensor becomes. The polysilicon sheet resistance for the temperature sensors is 8.3 Ω/sq [42] at room temperature. The sheet resistance of 8.3 Ω/sq, the length of 1300 μm, and the width of 0.95 μm are substituted into Equation (4). The initial resistance for the temperature sensor is obtained, and the value is 11.36 kΩ. The resistance change of the temperature sensor is given by [41],

(5) $$R_{s}=R_{0}\left[1+\alpha_{TCR}\left(T_{s}-T_{i}\right)\right]$$

where *R*_0_ is the initial resistance for the temperature sensor, *α_TCR_* is the TCR (temperature coefficient of resistor) for the temperature sensor, *T_s_* is the temperature change, and *T_i_* is the initial temperature for the temperature sensor. According to Equation (5), the resistance change of the temperature sensor relies on the temperature change, initial resistance, and TCR for the temperature sensor. The more the temperature increases, the higher the resistance of the temperature increases. The TCR of the temperature sensor is 1500 ppm/°C [23,42]. The TCR of 1500 ppm/°C and the initial resistance of 11.36 kΩ are substituted into Equation (5). The resistance change for the temperature sensor is obtained. The evaluation of the resistance change for the temperature sensor is shown in Figure 5. The results show that the resistance of the temperature sensor at room temperature is 11.36 kΩ. When the temperature increases to 373 K, the resistance of the temperature sensor becomes 12.72 kΩ.

## 3. Fabrication of the Thermoelectric Energy Micro Harvester

The thermoelectric energy micro harvester was manufactured utilizing the TSMC (Taiwan Semiconductor Manufacturing Company, Hsinchu, Taiwan) 0.18 μm CMOS (complementary metal oxide semiconductor) process. In addition to the CMOS process, the TEMH required a post-process because the TEMH required a suspended thermocouple structure. The 0.18 μm CMOS process contains one polysilicon layer and six metal layers. The material of the metal layers is aluminum. The thermocouples in the TEMH consist of n-type and p-type polysilicon layers. The hot part of the thermocouple is fixed on the silicon substrate. The cold part of the thermocouple is a suspended structure with cooling sheets. The cooling sheets are made up of a stacked structure that is composed of six metal layers, and the cooling sheets are used to enhance the heat dissipation. An integrated circuit software, Cadence, was employed to design the layout of the TEMH. According to the structure in Figure 1, the layout of the TEMH was established; then, TSMC carried out the fabrication of the TEMH using the CMOS process using this layout.

**Figure 6:**
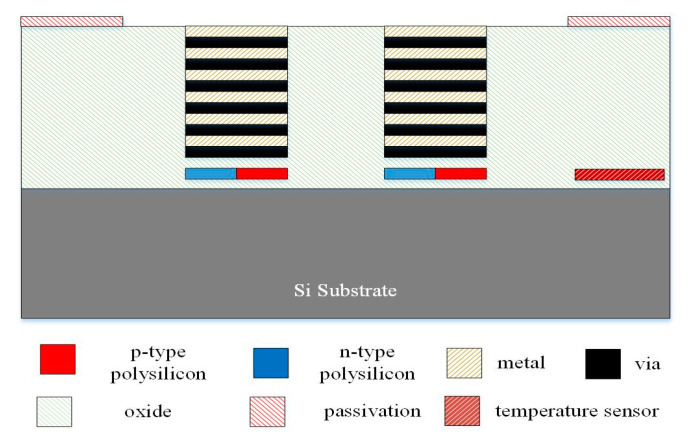
Cross section of the TEMH after the CMOS process.

**Figure 7:**
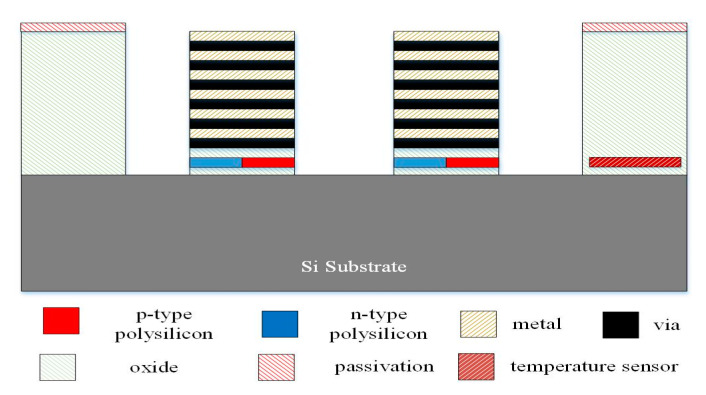
Cross section of the TEMH after etching of the silicon oxide layer.

**Figure 8:**
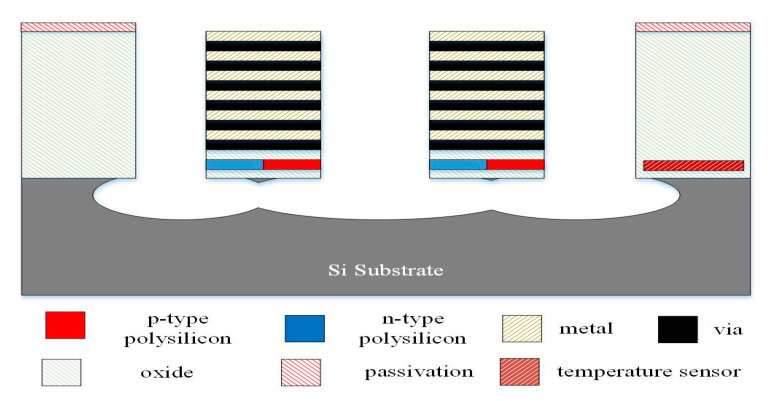
Cross section of the TEMH after etching of the silicon substrate.

Figure 6, Figure 7 and Figure 8 illustrate the process flow of the TEMH. Figure 6 presents a cross section of the TEMH along the AA line (Figure 1) after the CMOS process. As shown in Figure 6, the TEMH chip contained the thermocouples and temperature sensors. The temperature sensor was made of polysilicon in the CMOS process. To enhance the TD of the CHP for the thermocouples, the thermocouples must be made as a suspended structure. A post-process was utilized to etch the sacrificial materials to obtain the suspended thermocouple structure. In the TEMH, there are two sacrificial materials. One is silicon dioxide, and the other is silicon substrate. Figure 7 presents a cross section of the TEMH after the etching of the silicon oxide layer. The sacrificial silicon oxide is positioned between the polysilicon strips of the thermocouples. In the post-process, an RIE (reactive ion etch) was utilized to etch the sacrificial silicon oxide until the silicon substrate was exposed. The source of CF_4_/O_2_ was supplied into the RIE to etch the silicon oxide. The etching conditions were RF power 150 W, pressure 20 mTorr, gas flow CF_4_ 20 sccm, with O_2_ 1 sccm. Figure 8 presents a cross section of the TEMH after etching of the silicon substrate. The sacrificial silicon substrate is positioned under the cold part of the thermocouple. In the post-process, an RIE was employed to remove the sacrificial silicon substrate until the cold part of the thermocouples was suspended. The source of SF_6_/O_2_ was supplied into the RIE to etch the silicon substrate. The etching conditions were RF power 350 W, pressure 15 mTorr, gas flow SF_6_ 28 sccm, with O_2_ 4 sccm. As shown in Figure 8, the RIE SF_6_/O_2_ etched silicon was an isotropic etching.

**Figure 9:**
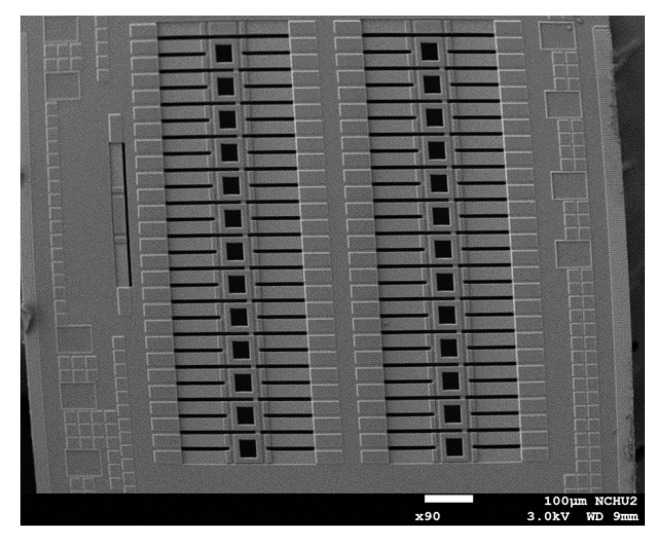
TEMH image taken by SEM.

**Figure 10:**
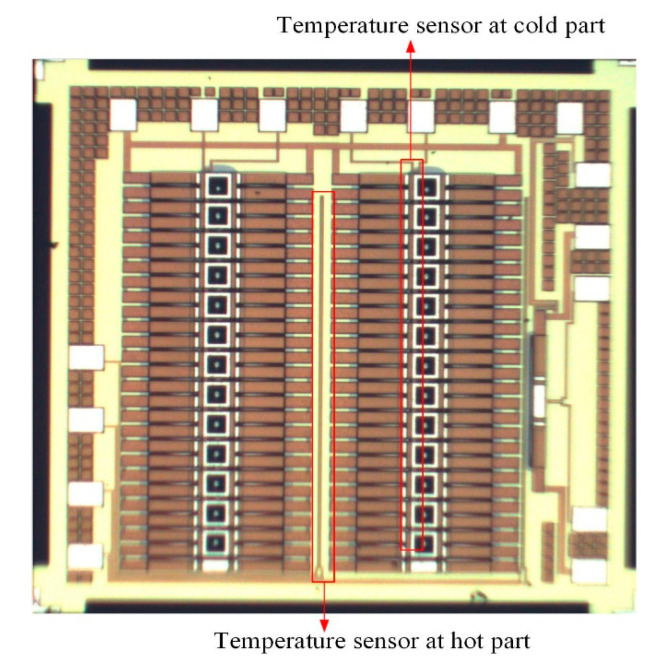
The TEMH taken by optical microscope.

**Figure 11:**
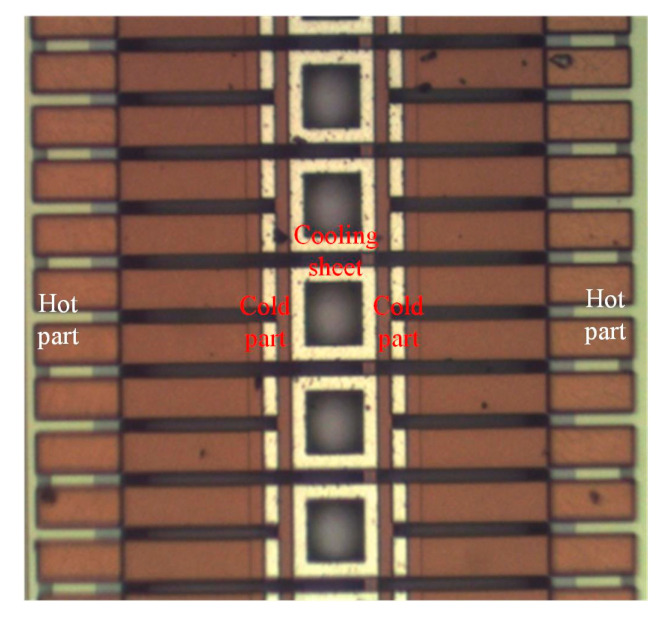
A partial image of the TEMH taken by an optical microscope.

An SEM (scanning electron microscope, NCHU, Taichung, Taiwan) was employed to measure the profile and structure of the TEMH. Figure 9 shows the profile of the TEMH taken by the SEM. As shown in Figure 9, the structure of the TEMH is complete, and the structure of thermocouples and the gap between the thermocouples can be clearly viewed. Figure 10 demonstrates the view of the TEMH taken by an optical microscope. As shown in Figure 10, the temperature sensors are respectively set near by the CHP of the thermocouples. Figure 11 shows a partial image of the TEMH taken by an optical microscope. As shown in Figure 11, the brown color in the picture shows the polysilicon strips of the thermocouples, which are regular and repeated structures.

**Figure 12:**
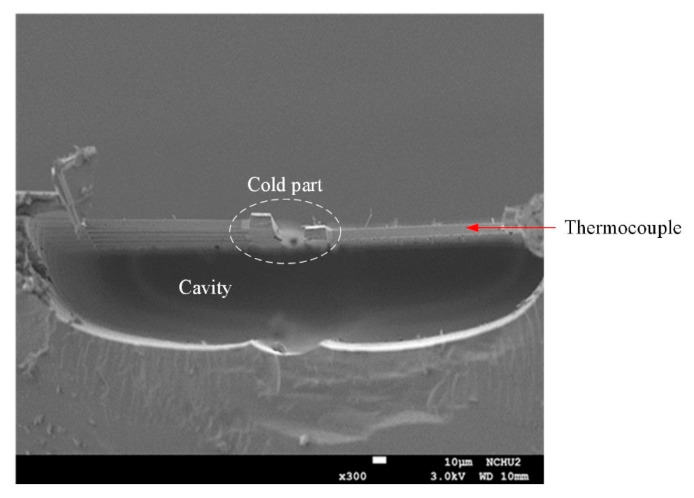
Cross-sectional image of the TEMH taken by SEM.

**Figure 13:**
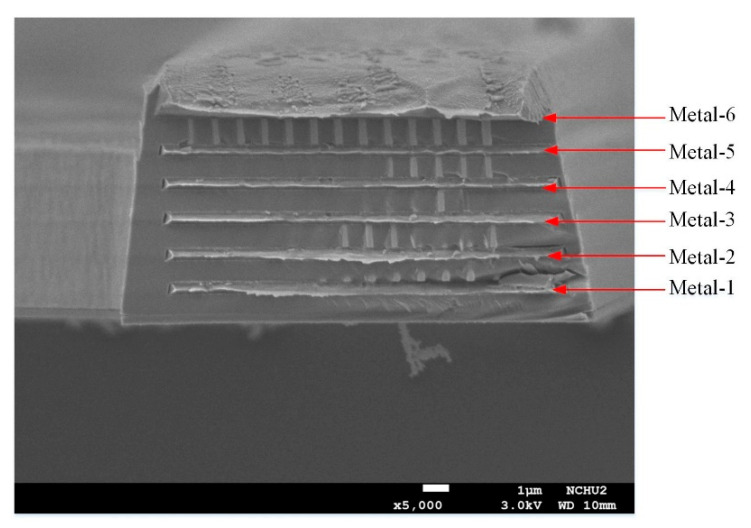
A cooling sheet image taken by SEM.

To determine if the cold part of the thermocouple was suspended, the cross-sectional structure of the TEMH was measured utilizing the SEM. Figure 12 shows cross-sectional image of the TEMH taken by the SEM. As shown in Figure 12, the cold part of the thermocouple is suspended, and it has a large cavity under the thermocouple. This means that the sacrificial silicon substrate is completely removed through the post-process, and the thermocouples have not incurred any damage during the post-process. To enhance heat dissipation, the cooling sheets were designed and mounted on the cold part of the thermocouples. A cooling sheet was measured utilizing the SEM to observe its structure. Figure 13 shows a cross-sectional image of a cooling sheet taken by the SEM. As shown in Figure 13, the cross-sectional structure of the cooling sheet, which is composed of metal-1 to metal-6, is clearly visible.

**Figure 14:**
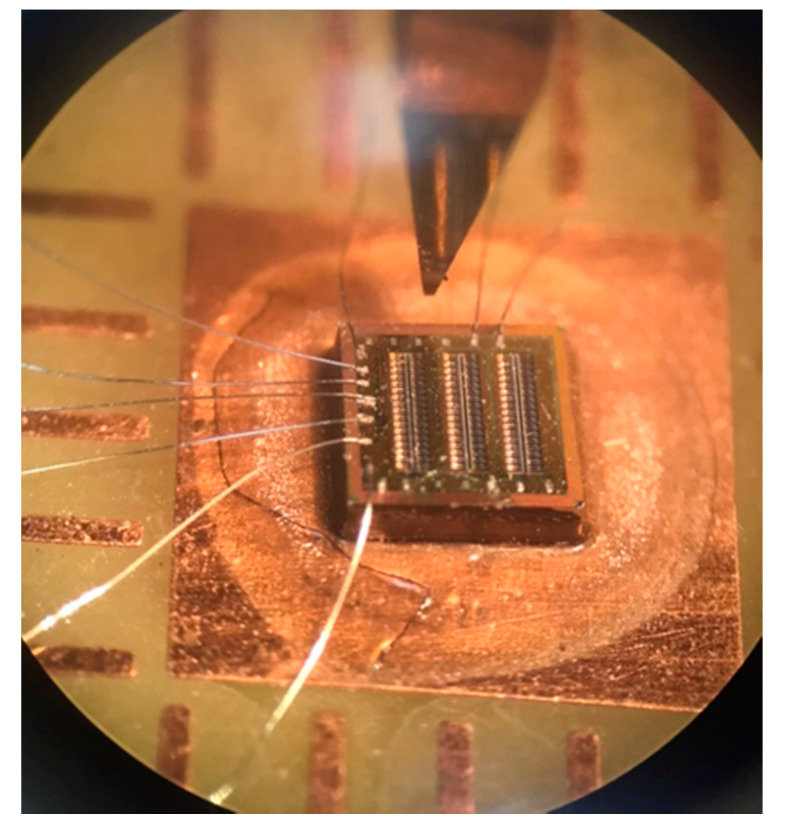
Wire bonding for the TEMH.

Finally, the performance of the TEMH must be tested. Before testing, the TEMH chip must be wire bonded. Figure 14 shows an image of wire bonding for the TEMH chip. The TEMH was mounted on a PCB (printed circuit board), and a wire bonder was utilized to connect the pads of the TEMH chip to the PCB.

## 4. Results

**Figure 15:**
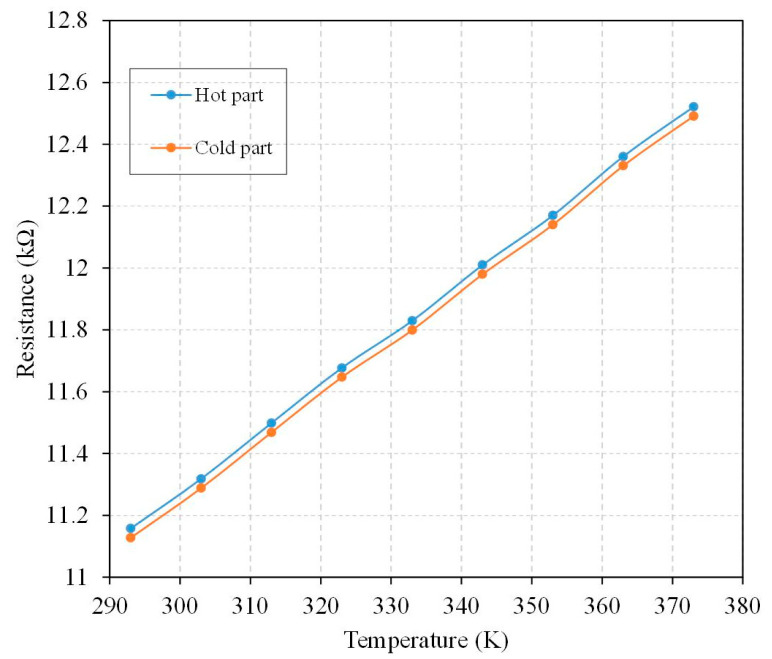
Measurement of resistance for the temperature sensors under various temperatures.

There were two kinds of temperature sensors in the thermoelectric energy micro harvester. One was mounted near the hot part of the thermocouples, and the other was mounted near the cold part of thermocouples. The temperature sensors could monitor the temperature for the CHP of the thermocouples in real time. To characterize the performance of the temperature sensors, a heat chamber was utilized to test the temperature sensors. The heat chamber could generate a heat source and provide a testing temperature to the temperature sensors. First, the TEMH chip was mounted in the heat chamber. Then, the output signal ports of the temperature sensors were connected to a digital multimeter, which was utilized to measure the output for the temperature sensors. When the heat chamber supplied a testing temperature to the temperature sensors, the sensor produced a change in resistance. The resistance of the temperature sensors was measured utilizing the digital multimeter. Figure 15 shows the measurement of resistance for the temperature sensors under various temperatures. As shown in Figure 15, the resistance change of the temperature sensor at the hot part of the thermocouples is linear, and the slope of the curve is 0.017 kΩ/K. The resistance change of the temperature sensor at the cold part of the thermocouples is also linear, and the slope of the curve is 0.017 kΩ/K. The two kinds of temperature sensors used a linear output and had the same slope of curve.

**Figure 16:**
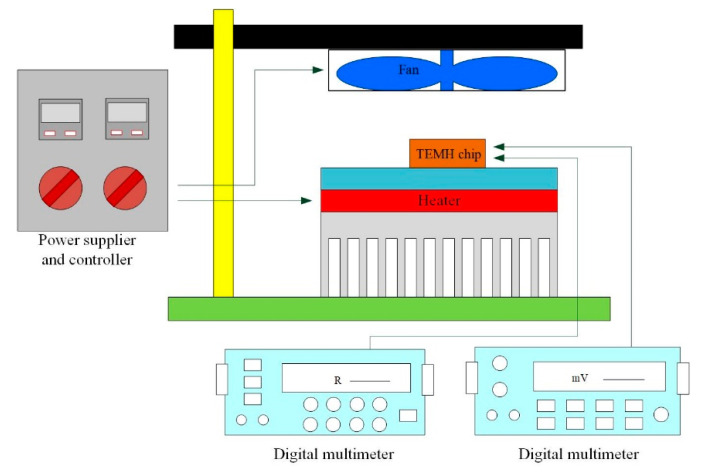
Measurement setup for the TEMH.

To understand the actual performance of the thermoelectric energy micro harvester, a set of experimental devices were established for testing the TEMH. Figure 16 shows the experiment setup for testing the actual performance of the TEMH. The experiment needed to have a controller, a heater, a fan, and digital multimeters. The controller employed to control the heater and the fan could tune the heating of the heater and the speed of the fan. The heater provided the heat source, and the fan supplied the cooling source required by the TEMH. The digital multimeters were utilized to detect the resistance of the temperature sensors and the output voltage of the TEMH. First, the TEMH chip was mounted in the experimental device, as shown in Figure 16. Then, the output ports of the temperature sensors and the TEMH were connected to the digital multimeters. When the heat source of the heater conducted to the TEMH and the CHP of the thermocouples obtained a TD, the TEMH generated an output voltage that was recorded by utilizing the digital multimeter. The temperature at the CHP of the thermocouples was monitored by utilizing the temperature sensors.

**Figure 17:**
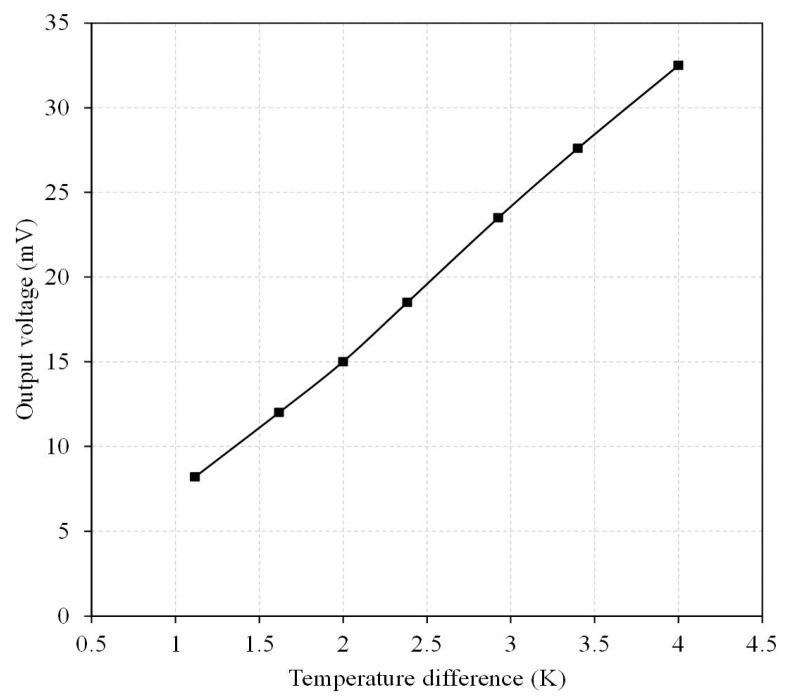
Measured results of output voltage for the TEMH.

The output voltage of the TEMH was measured. Figure 17 shows the measured results of output voltage for the TEMH. The results revealed that the TEMH had an output voltage of 8.2 mV, as the TD was 1.1 K. When the TD increased to 4 K, the output voltage became 32.5 mV. The slope of the curve in Figure 17 was 8.57 mV/K, so the output voltage factor of the TEMH was 8.93 mV/mm^2^K. Compared to the simulated results, the measured output voltage factor for the TEMH had an error percentage of 5%.

**Figure 18:**
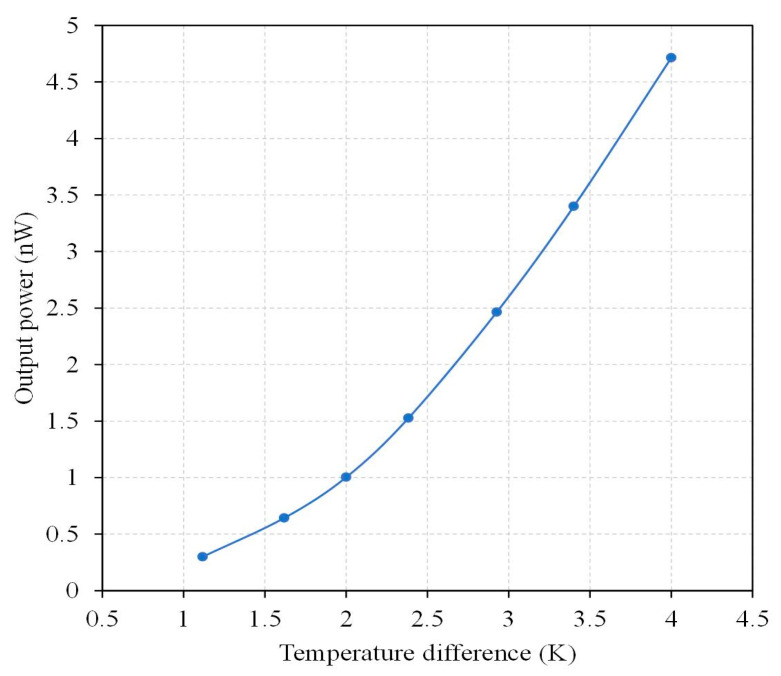
Measured results of maximum output power for the TEMH.

According to the measurement results, the thermoelectric energy micro harvester had an internal resistance of 56.5 kΩ. The output power of the TEMH could be obtained through Equation (2). The measured output voltage in Figure 17 and the internal resistance of 56.5 kΩ were substituted into Equation (2). The maximum output power of the TEMH was obtained. Figure 18 presents the measured results of maximum output power for the microgenerator. The results revealed that the TEMH had a maximum output power of 0.3 nW, as the TD was 1.1 K. When the TD increased to 4 K, the maximum output power became 4.72 nW. The area of the TEMH was 0.96 mm^2^. The measured power factor of the TEMH was 0.31 nW/mm^2^K^2^. The evaluated power factor for the TEMH was 0.36 nW/mm^2^K^2^. Compared to the evaluated power factor, the measured power factor for the TEMH had an error percentage of 13%. This is due to that was the variation of the thermoelectric coefficient for the thermocouples which resulted from the doping process, creating an error in the output voltage factor and the power factor of the TEMH. 

Peng [19] developed a TEMH that had an output voltage factor of 0.178 mV/mm^2^K and a power factor of 1.47 × 10^−3^ pW/mm^2^K^2^. A TEMH presented by Zhang [20] had an output voltage factor of 0.58 V/cm^2^K and a power factor of 2.76 × 10^−2^ μW/cm^2^k^2^. Xie [22] fabricated a TEMH with the power factor of 0.052 μW/cm^2^K^2^. Yang [23] proposed an in-plane TEMH in which the output voltage factor and power factors were 4.423 V/cm^2^K and 0.0473 μW/cm^2^K^2^, respectively. Sun [24] manufactured a hybrid TEMH. The output voltage factor of the TEMH was 0.316 V/cm^2^K and the power factor of the TEMH was 6.34 × 10^−3^ μW/cm^2^K^2^. A flexible TEMH, developed by Glatz [25], had a power factor of 0.83 μW/cm^2^K^2^. Huesgen [26] made a TEMH with a power factor of 3.63 × 10^−3^ μW/mm^2^K^2^. A flexible TEMH, fabricated by Glatz [27], had a power factor of 0.29 μW/cm^2^K^2^. Kao [29] presented a TEMH with a power factor of 6.4 × 10^−2^ nW/cm^2^K^2^. In this work, the output voltage factor and power factor of the TEMH were 8.93 mV/mm^2^K and 0.31 nW/mm^2^K^2^, respectively. Compared to the TEMHs of Peng [19], Zhang [20], Xie [22], Yang [23], Sun [24], Huesgen [26], Glatz [27], and Kao [29], the power factor of the TEMH in this work exceeded that of Peng [19], Zhang [20], Sun [24], and Kao [29].

## 5. Conclusions

The thermoelectric energy micro harvester was successfully manufactured employing the commercial CMOS process. The TEMH was constructed using a series of 54 thermocouples. The hot part of the thermocouple was fixed on the silicon substrate. The heat source conducted heat to the thermocouple through the silicon substrate. The more the TD at the CHP of the thermocouples increased, the higher the output voltage and output power of the TEMH became. To obtain a higher output voltage and output power of the TEMH, the cold part of the thermocouples was fabricated as a suspended structure and combined with cooling sheets. The cooling sheets that were mounted above the cold part of the thermocouples could increase heat dissipation so that the TD at the CHP of the thermocouples increased. To form the suspended thermocouple structure, the TEMH required a post-process to remove the sacrificial materials. The post-process involved two steps. One was to etch the sacrificial silicon dioxide utilizing RIE CF_4_/O_2_, and the other was to etch the sacrificial silicon substrate using SF_6_/O_2_. The results showed that the structure of the thermocouples was completely suspended and did not exhibit any injury. The measured results revealed that the TEMH had an output voltage of 32.5 mV when the TD at the CHP of the thermocouples was 4 K. The TEMH had a voltage factor of 8.93 mV/mm^2^K. The maximum output power of the TEMH was 4.72 nW when the TD at the CHP of the thermocouples was 4 K. The TEMH had a maximum power factor of 0.31 nW/mm^2^K^2^.

## Figures and Tables

**Table 1:** Performances of THMEs.

Authors	Voltage Factor (mV/mm^2^K)	Power Factor (nW/mm^2^K^2^)
Peng [19]	0.178	0.00147
Zhang [20]	5.8	0.276
Xie [22]	‒	0.52
Yang [23]	44.23	0.473
Sun [24]	3.16	0.0634
Glatz [25]	‒	8.3
Huesgen [26]	‒	3.63
Glatz [27]	‒	2.9
Kao [29]	‒	0.00064

**Table 2:** Thermal conductivity of materials.

Material	Silicon	Aluminum	Polysilicon	Silicon Dioxide
Thermal conductivity (W/m·K)	150	236	31.5	1.42
